# Supplementary material for: The interactome of intact mitochondria by cross-linking mass spectrometry provides evidence for coexisting respiratory supercomplexes
Source: Mol Cell Proteomics. 2017 Dec 8;17(2):216–32. doi: 10.1074/mcp.RA117.000470 (PMC5795388; doi:10.1074/mcp.RA117.000470)
Supplement: Supplemental Data [file supp_RA117.000470_133922_0_supp_23978_fzffwf.zip › spectra_annotation/mito_DR_spectra_annotation/252-1-17-1-120-1.pdf]

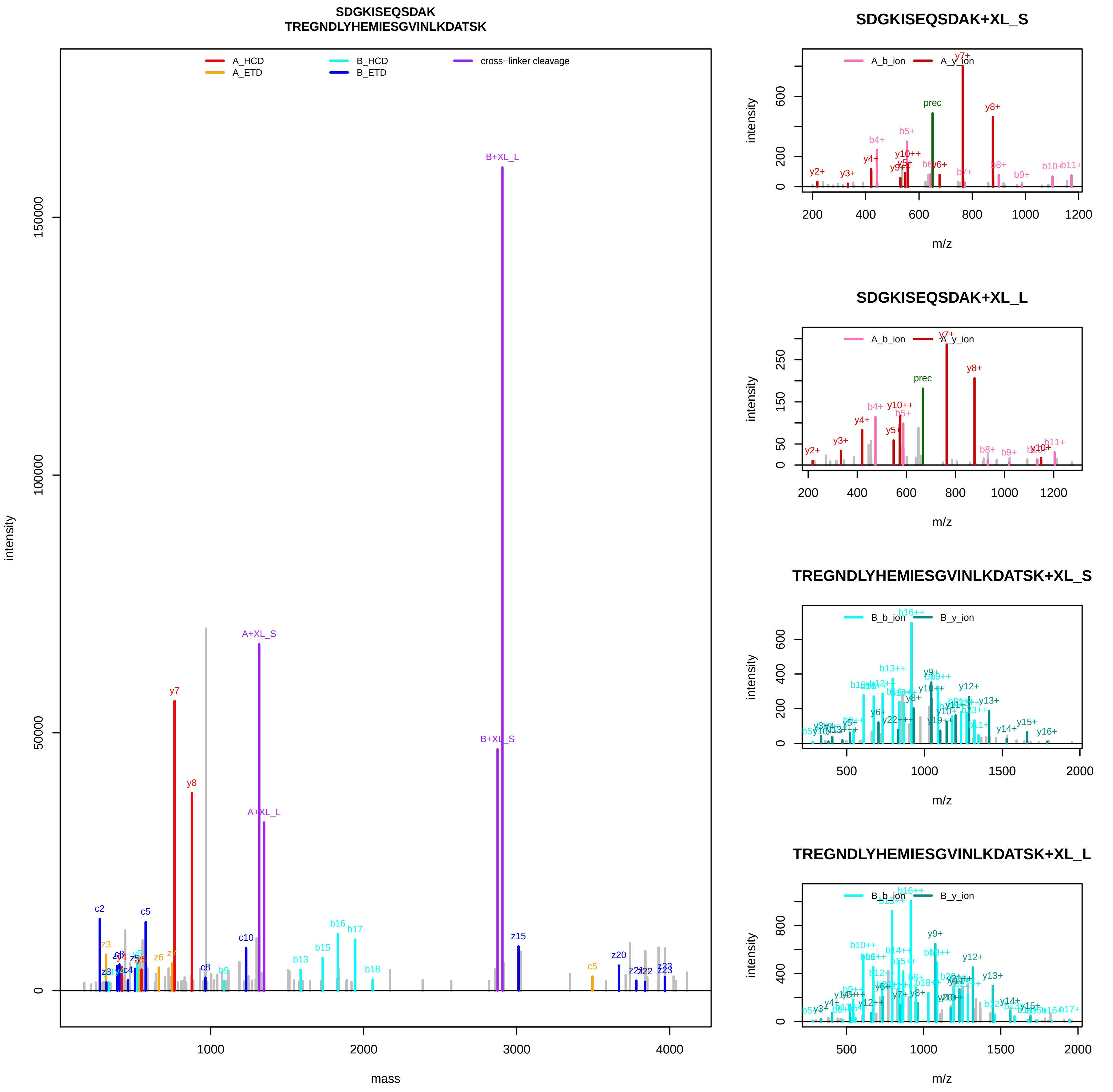
SDGKISEQSDAK
TREGNDLYHEMIESGVINLKDATSK
A_HCD
A_ETD
B_HCD
B_ETD
cross−linker cleavage
B+XL_L
A+XL_S
B+XL_S
A+XL_L
y7
y8
c2
c5
c8
c10
b13
b15
b16
b17
b18
z15
z20
c5
z23
intensity
mass
0
50000
100000
150000
1000
2000
3000
4000
SDGKISEQSDAK+XL_S
A_b_ion
A_y_ion
prec
y7+
y8+
b5+
b4+
y4+
y10++
y2+
y3+
b9+
b10+
b11+
intensity
m/z
SDGKISEQSDAK+XL_L
A_b_ion
A_y_ion
prec
y7+
y8+
b4+
y10++
y4+
y3+
y2+
b8+
b9+
b11+
intensity
m/z
TREGNDLYHEMIESGVINLKDATSK+XL_S
B_b_ion
B_y_ion
b16++
b13++
y9+
y12+
y13+
y14+
y15+
y16+
intensity
m/z
TREGNDLYHEMIESGVINLKDATSK+XL_L
B_b_ion
B_y_ion
b16++
b13++
y9+
y12+
y13+
b10++
b17+
intensity
m/z
